# Plasma Metabolomics Identifies the Dysregulated Metabolic Profile of Primary Immune Thrombocytopenia (ITP) Based on GC-MS

**DOI:** 10.3389/fphar.2022.845275

**Published:** 2022-05-24

**Authors:** Ziyan Zhang, Xiaojin Wu, Meng Zhou, Jiaqian Qi, Rui Zhang, Xueqian Li, Chang Wang, Changgeng Ruan, Yue Han

**Affiliations:** ^1^ National Clinical Research Center for Hematologic Diseases, Jiangsu Institute of Hematology, The First Affiliated Hospital of Soochow University, Suzhou, China; ^2^ Institute of Blood and Marrow Transplantation, Collaborative Innovation Center of Hematology, Soochow University, Suzhou, China; ^3^ Key Laboratory of Thrombosis and Hemostasis of Ministry of Health, Suzhou, China; ^4^ State Key Laboratory of Radiation Medicine and Protection, Soochow University, Suzhou, China

**Keywords:** primary immune thrombocytopenia, gas chromatography tandem mass spectrometry, metabolomics, OPLS -DA, metabolomics pathway analysis

## Abstract

ITP is a common autoimmune bleeding disorder with elusive pathogenesis. Our study was implemented to profile the plasma metabolic alterations of patients diagnosed with ITP, aiming at exploring the potential novel biomarkers and partial mechanism of ITP. The metabolomic analysis of plasma samples was conducted using GC-MS on 98 ITP patients and 30 healthy controls (HCs). Age and gender matched samples were selected to enter the training set or test set respectively. OPLS-DA, t-test with FDR correction and ROC analyses were employed to screen out and evaluate the differential metabolites. Possible pathways were enriched based on metabolomics pathway analysis (MetPA). A total of 85 metabolites were investigated in our study and 17 differential metabolites with diagnostic potential were identified between ITP patients and HCs. MetPA showed that the metabolic disorders of ITP patients were mainly related to phenylalanine, tyrosine and tryptophan biosynthesis, phenylalanine metabolism and glyoxylate and dicarboxylate metabolism. Additionally, we discriminated 6 differential metabolites and 5 enriched pathways in predicting the resistance to glucocorticoids in chronic ITP patients. The distinct metabolites discovered in our study could become novel biomarkers for the auxiliary diagnosis and prognosis prediction of ITP. Besides, the dysregulated pathways might contribute to the development of ITP.

## Introduction

ITP is a common hematological disease, accounting for about one-third of clinical hemorrhagic disease. The diagnostic criteria of ITP are still based on clinical exclusion method, excluding thrombocytopenia secondary to other factors (such as infection, drugs and various connective tissue diseases, etc.) (Neunert et al., 2020). Treatment with glucocorticoids is recommended as the standard first-line strategy, achieving responses in 50–85% of ITP patients (Mithoowani et al., 2016; Y.; Kong et al., 2018). However, nearly 80% of the adult patients fail or become dependent on corticosteroids and require subsequent therapy like TPO receptor agonists, rituximab, splenectomy, etc (Ghanima et al., 2021). Till now, the pathogenesis of ITP remains obscure, antibody-mediated platelet destruction, T cell imbalance (Li J. et al., 2020), platelet desialylation (Li J. et al., 2015) and oxidative stress (Qi et al., 2017) have been recognized as important mechanisms of ITP.

In recent years, metabolomics obtained great development in disease diagnosis, pharmaceutical research, and other fields. The components of metabolome can be viewed as the end products of gene expression in a particular physiological period or physiological conditions and define the biochemical phenotype of a cell or tissue. These products are small molecule compounds involved in metabolism, maintenance of normal function and development of the organism, mainly including some endogenous molecules with relative molecular weight less than 1,000 daltons (Sumner et al., 2003). Metabolomics is a powerful tool for investigating phenotypic abnormalities in mutant animals and human diseases, and in modelling physiological variation in experimental animals and man (Nicholson et al., 1999). Gas chromatography tandem mass spectrometry (GC-MS) is an analytical instrument for measuring the mass to charge ratio (m/z) of ions, with the advantages of high resolution, high flux, high precision, sensitivity and high reproducibility, and can determine some heat-resistant metabolites, which is suitable for the analysis of substances with small polarity (Jonsson et al., 2005).

Benefitting from advances in omics technologies, metabolomics methods have been widely used to explore potential markers of diseases and gain novel understanding of the metabolic pathway changes in pathogenesis. At present, metabolomics methods have been applied in the study of various hematological malignancies (Chanukuppa et al., 2018), diabetes mellitus (Arneth et al., 2019), hypertension, coronary heart disease (McGarrah et al., 2018) and others. The extensive use of metabolomics methods has also attracted high attention in the study of the physiological activities of platelets, and several studies have been implemented to elucidate metabolic changes of platelets in resting or activated states. (Aibibula et al., 2018) found that platelets switched freely between glycolysis and OXPHOS, consuming either glucose or fatty acids at rest. The transition of platelets from a quiescent to an activated state promoted rapid uptake of exogenous glucose, and preferentially utilize glycolysis for ATP generation when activated. Dhenge et al. found that arachidonic acid (AA)/DHA appears to have enhanced megakaryocytes and platelets generation in cell numbers, surface marker expression, cellular ploidy and expression of cytoskeletal components (Dhenge et al., 2017). Li et al. reported that indoleamine 2, 3-dioxygenase (IDO) mediates abnormal tryptophan metabolism and plays an important role in the pathogenesis of ITP (Li Z. J. et al., 2015). Besides, branched-chain amino acids (BCAA) metabolism was reported to regulate platelet activation and was associated with arterial thrombosis risk (Y. Xu et al., 2020). To our knowledge, only few studies have investigated ITP with the implement of metabolomics so far. A study concerning gut microbiome and metabolome showed that several ITP-altered gut bacteria as well as metabolites could be diagnostic biomarkers for ITP, and were highly correlated with platelet counts, suggesting the potential role of metabolomics methods in the pathogenesis mining of ITP (Zhang X. et al., 2020).

In this study, we developed a stable and reliable GC-MS analytical method to detect human plasma samples from ITP patients, expecting to identify the plasma metabolomic profile of ITP. We screened out differential metabolites and metabolic pathways related to ITP to provide new indexes for diagnosis and prognosis of ITP. Our finding could provide a new approach to reveal possible mechanism of ITP.

## Patients and Methods

### Enrollment of Patients and Diagnosis Criteria

ITP patients who were hospitalized at the First Affiliated Hospital of Soochow University from August 2018 to April 2019 were enrolled in this study at the ratio of 3:1 for ITP group and HC group respectively with age and gender matched. In order to further understand the changes of metabolites in ITP patients before and after treatment, the plasma samples of patients before and till the platelet count of 100 × 10^9^/L after treatment were collected, and the metabolites were detected by GC-MS method.

The diagnosis and staging of ITP were based on the consensus of Chinese experts on the diagnosis and treatment of adult primary immune thrombocytopenia (2020) (Thrombosis and Hemostasis Group, Chinese Society of Hematology, Chinese Medical Association, 2020). Newly diagnosed ITP (nITP) is defined as up to 3 months from diagnosis. The course of disease of persistent ITP (pITP) is 3–12 months, and chronic ITP (cITP) is defined as ITP lasting more than 12 months (Neunert et al., 2019). Sustained remission (SR) was defined as maintenance of platelet count above 50 × 10^9^/L for over 6 months (Ahn and Harrington, 1977)and patients did not reach the standard were defined as non-SR (NSR). The good therapeutic effect is defined as a platelet count over 30 × 10^9^/L independent of transfusion, or at least twice the baseline platelet level of the patient without any bleeding symptoms after treatment. Patients that don’t reach the criteria above are considered poor therapeutic effect and need to switch to second-line treatment (Thrombosis and Hemostasis Group, Hematology Society, Chinese Medical Association, 2016).

### Study Approval

The study protocol was approved by the Faculty Hospital Ethics Committee at the First Affiliated Hospital of Soochow University in accordance with the guidelines in the Declaration of Helsinki. All ITP patients and HCs in this study signed informed consent forms, requiring patients and HCs to have a light diet and avoid stimulation from tobacco, alcohol and spicy food before sampling, so as to reduce the fluctuation of metabolite levels caused by dietary factors. Healthy subjects matched with the age and gender of ITP patients were selected to minimize the possible interference factors.

### Sample Acquisition and Preservation

All fasting peripheral blood samples were collected with EDTA anticoagulation tubes and centrifuged at 3000 rpm for 15 min. Each plasma sample was divided into equal aliquots and stored at −80°C for analysis.

With batch experiments, plasma samples were thawed at room temperature, 100 μl of which were extracted and vortexed for quality control (QC). Subsequently, another 100 μl plasma was taken and mixed with 400 μl glacial acetonitrile, 80 μl 2, 4-dichlorobenzoic acid (BOC) with concentration of 0.2 mg/ml and 80 μl tridecanoic acid with concentration of 5 μg/ml as the internal standard, and then vortexed and mixed for 2 min. Then ultrasonic extraction was carried out for 15 min (1°C), followed by centrifugation for 15 min (13000 rpm/min, 4°C), and the supernatant 450 μl was transferred to a new EP tube and placed in a refrigerator at −80°C for more than 6 h. Then the samples were made into freeze-dried powder to ensure that the sample is absolutely anhydrous. The dried samples were redissolved with methoxyamine pyridine solution (15 mg/ml, 50 μl) and vortexed. Afterwards, the sample was oximated in a metal bath thermostat at 70°C for 1 h, followed by mixture with 50 μl N-methyl trimethylsilyl trifluoroacetyl (MSTFA) [containing 1% trimethylchlorosilane (TMCS) as catalyst] in a metal bath thermostat at 70°C for 1 h and cooling for half an hour. Finally, the derivatized samples were mixed with 100 μl n-heptane and centrifuged (13000 rpm/min, 4°C, 15 min) and then the supernatant was analyzed by GC-MS. All derived samples were tested by GC-MS within 2 days after derivation to ensure the stability and reliability of the results.

### Gas Chromatography-Mass Spectrometry -Based Metabolomics

Metabolic profiling of plasma samples was acquired using a TSQ 8000 EVO Triple Quadpole GMS (Thermo, United States). Each derivatized sample was injected into a DB-5 fused silica capillary column (30 m × 0.25 mm ×0.25 μM; Agilent, United States) in a split mode (ratio 10:1). Helium with high purity (99.9%) was used as the carrier gas, and operated with a constant flow rate of 1.1 ml/min. The initial column temperature of 80°C was maintained for 5 min, then increased to 170°C at 5°C/min, and finally to 300°C at 10°C per minute intervals. The temperatures of the inlet and ion source were at 280 and 230°C. The mass spectra data were obtained in a full scan with 30–600 m/z. Plasma samples were all running in a random order and the QC sample was inserted approximately every 10 experimental samples.

The plasma metabolites were analyzed by GC-MS, and the original data were imported to obtain the Total Ion Chromatogram (TIC). Automated Mass Spectral Deconvoltion and Identification System (AMDIS) and the National Institute of Standards Technology (NIST) 11.0 mass spectrometry database for mass spectrometric detection, deconvolution, identification and matching of each chromatographic peak and combined with the standard and retention time to further identify the substance.

### Statistical Analysis

After the metabolites in plasma were identified, the original data were processed in the XCalibur workstation, and the chromatographic peaks were integrated according to retention time and quality. The integral results were corrected using the internal standard (2,4-dichrobenzoic acid). Finally, the corrected area ratio of each metabolite was obtained to form the metabolite matrix.

In order to find the characteristic metabolites of ITP patients, propensity score matching (PSM) was conducted to match patients on age and gender between the two groups, entering the training set and test set respectively. In the training set, orthogonal partial least squares discriminant analysis (OPLS-DA) and t-test combined with false discovery rate (FDR) correction were used to screen out the differential metabolites of ITP patients and HC. The receiver operating curve (ROC) was applied to evaluate the discriminatory capability of the differential metabolites in the training set and the test set respectively. Univariate and multivariate logistic regression analyses were performed to identify predictive factors for treatment and prognosis. Finally, MetPA was implemented to construct the metabolic pathway in MetaboAnalyst 5.0. OPLS-DA model was also carried out to compare the plasma metabolomic profiles of the subgroups of ITP patients to further explore factors concerning clinical application.

## Results

### Characteristics of Immune Thrombocytopenia Patients

Ninety-eight ITP patients hospitalized at the First Affiliated Hospital of Soochow University from August 2018 to April 2019 were enrolled in this study, including 29 males and 69 females, with a median age of 42.5 years (12–72 years). 30 HCs in the same period were selected as the control group, including 10 males and 20 females, with a median age of 39.5 years (14–78 years). Data from 114 plasma samples (from 57 patients on admission and after treatment) was further collected to validate the differential metabolites screened from ITP patients. Main characteristics of the study participants are summarized in Table 1.

**TABLE 1:** Clinical characteristics of the study participants.

Characteristics	ITP patients	Controls	*p* value
All	98	30	
Sex			0.679
Male	29 (29.6%)	10 (33.3%)	
Female	69 (70.4%)	20 (66.7%)	
Age			0.289
Median	42.5	39.5	
Range	14–72	14–78	
Clinical stages			
Newly diagnosed ITP (nITP)	44 (44.9%)		
Persistent ITP (pITP)	11 (11.2%)		
chronic ITP (cITP)	43 (43.9%)		
Therapeutic effect			
Good	62 (63.3%)		
Poor	36 (36.7%)		

2:1 PSM was conducted in the case group (98 patients with ITP) and the control group (30 HCs) according to gender and age, certain factors that may influence the plasma metabolomic profile (Supplementary Table S1) (Laugsand et al., 2016; Han et al., 2021). As a result, a total of 30 ITP patients and 15 HCs were successfully matched into the training set, while the remaining 68 ITP patients and 15 HCs samples entered the test set.

### Comprehensive Metabolomic Analysis of Immune Thrombocytopenia Patients

**FIGURE 1:**
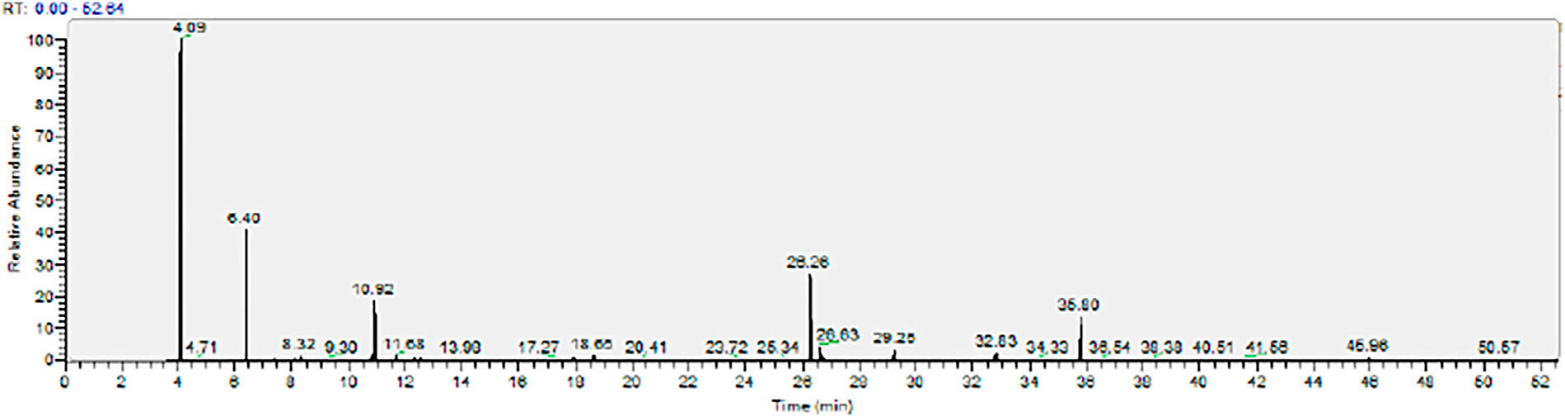
Representative TIC of the plasma samples.

The metabolomic analysis of plasma samples was conducted using GC-MS on 98 ITP patients and 30 HCs. Quality control (QC) samples were prepared in parallel according to the pretreatment method of plasma samples in the experimental group to monitor the reproducibility of experimental sample preparation. Representative GC-MS total ion chromatograms (TIC) over the QC plasma sample is shown in Figure 1. AMDIS and NIST mass spectrometry databases were used for mass spectrometric detection, deconvolution, identification and matching of each chromatographic peak, and retention time (RT) and mass charge ratio were combined to identify the chromatographic peaks. The retention time drift of each chromatographic peak was limited to 30 s. Then, 85 metabolites were finally identified, including saccharides, amino acids, fatty acids and so on.

### Immune Thrombocytopenia Exhibited a Particular Metabolomic Profile With Diagnostic Potential

**FIGURE 2:**
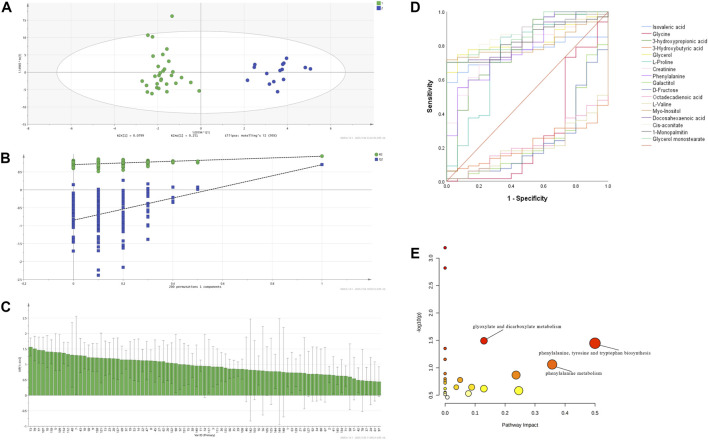
ITP exhibited a particular metabolomic profile with diagnostic potential. OPLS-DA model based on the data from the ITP patients and HCs **(A)** score scatter plot **(B)** 200 random permutation tests **(C)** VIP plot **(D)** ROC of the 17 differential metabolites with biomarker potential in ITP in the test set **(E)** MetPA of differential plasma metabolites in ITP patients.

In order to better visualize and denoise the multidimensional data, orthogonal partial least squares discriminant analysis (OPLS-DA) was applied to model the difference between the ITP patients and HCs in the training set and the parameters of the score plot R^2^Y = 0.937 and Q^2^ = 0.701 (Figure 2A) suggested the good explanatory and predictive ability of our model, respectively. The OPLS-DA model was further validated by the 200 random permutation tests with Q^2^ = -0.847 (Q^2^ < 0.05), indicating the model was not over-fitting (Figure 2B). Distinct separation was observed between ITP patients and HCs in the score scatter plot based on the OPLS-DA supervised model and 36 metabolites with a variable importance in projection (VIP) value > 1 (Sinclair et al., 2021) were chosen as differential biomarkers initially (Figure 2C). In combined with t-test and false discovery rate (FDR) correction, we identified 32 metabolites with *p* < 0.05 that may have potential to distinguish ITP patients from HCs (Supplementary Table S2). ROC analysis was performed to evaluate the differential metabolites in the training set and the test set separately. The area under curve (AUC) of each differential metabolites was calculated to determine their individual performance as ITP diagnostic biomarkers. In the training set, 23 metabolites were selected with an AUC > 0.7, followed by the ROC analysis conducted in the test set as external verification. As a result, 17 metabolites with an AUC > 0.7 (Figure 2D and summarized in Supplementary Table S3) were identified as differential metabolites with significant diagnostic potential to distinguish ITP patients from HCs: glycerol monostearate, 1-monopalmitin, glycerol, DHA, octadecadienoic acid, isovaleric acid, cis-aconitate, D-fructose, galactitol, myo-inositol, L-valine, phenylalanine, L-proline, glycine, 3-HB, creatinine, 3-hydroxypropionic acid.

The distinct variation of metabolomic profile between ITP patients and HCs prompted us to explore metabolites that could be used to predict prognosis. To determine which metabolites were of significant prognostic value in ITP patients, 17 differential metabolites and related clinical characteristics such as gender, age, clinical stage, severity, therapeutic effect of glucocorticoids, Helicobacter pylori (H. pylori) infection, reticulocyte, platelet-associated immunoglobulin (PAIgG) were compared among ITP patients between sustained remission (SR) group and non-SR (NSR) group. Univariate analysis showed that glycerol, 3-hydroxypropionic acid, galactitol, D-fructose, cis-aconitate (*p* < 0.05) were significantly associated with the NSR. Only metabolites with *p* < 0.2 in the univariate analysis were further analyzed in the multivariate logistic regression model (Kang et al., 2013). The multivariate analysis identified that glycerol (OR = 1.049, 95%CI: 1.009–1.091) was independently associated with higher risk of NSR Table 2.

**TABLE 2:** Univariate and multivariate logistic regression model for the risk of NSR.

Metabolites	Univariate		Multivariate		
	*p* value	*p* value		OR	95%CI
Glycerol	0.014	0.017		1.049	1.000–1.091
3-hydroxypropionic acid	0.030				
Galactitol	0.002				
D-Fructose	0.019				
Cis-aconitate	0.015				
Clinical stage	0.023				

Using MetaboAnalyst 5.0 online platform, pathway analysis of 17 differential metabolites was conducted and the enriched pathways were screened out by the threshold of impact value > 0.05 and -log10(p) > 1. Three deregulated pathways in ITP were identified and annotated in the bubble plot (Figure 2E): phenylalanine, tyrosine and tryptophan biosynthesis, phenylalanine metabolism, glyoxylate and dicarboxylate metabolism, with pathway impact values of 0.5, 0.36, 0.13 and the -log10(p) value of each pathway was 1.45, 1.06, 1.49, respectively.

### The Metabolomic Profile did not Vary Significantly Before and After Treatment in Immune Thrombocytopenia Patients

To further explore the changes of metabolites in ITP patients before and after treatment, the principal component analysis (PCA) model was developed to compare the difference between the two groups. As was suggested in the score scatter plot below (Supplementary Figure S1), the metabolomic profile failed to show a separation tendency before and after treatment in ITP patients. Given the plasma samples were collected at the time of platelet counts just recovered and the limit of sample size, further researches are needed to determine the metabolomic profile of patients with stable remission. Besides, the metabolomic profile grouped by gender, age, clinical stage, severity, reticulocyte, PAIgG among the ITP patients in our study did not separate significantly.

### Six Differential Metabolites Were Identified From the cITP Patients Grouped by Therapeutic Effect of Glucocorticoids

**FIGURE 3:**
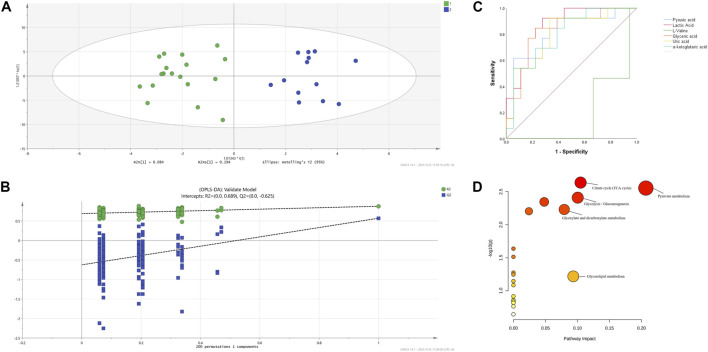
OPLS-DA model based on the data from the cITP patients grouped by therapeutic effect **(A)** score scatter plot **(B)** 200 random permutation tests **(C)** ROC of the six differential metabolites with biomarker potential **(D)** MetPA of differential plasma metabolites in cITP patients grouped by therapeutic effect.

The OPLS-DA supervised model was also used to compare plasma metabolomic profiles of cITP patients with good and poor therapeutic effect of glucocorticoids. The score scatter plot showed a clear distribution between the two groups (Figure 3A) and was validate by permutation tests (Figure 3B). The following metabolites with a VIP value > 1, *p* < 0.05 as well as an AUC > 0.7 were considered as metabolites with biomarker potential: lactic acid, glyceric acid, L-valine, pyruvic acid, uric acid, α-ketoglutaric acid, showing high accuracy and specificity in predicting the therapeutic effect of ITP patients with chronic phase. The AUC of the 6 differential metabolites were 0.868, 0.825, 0.816, 0.812, 0.803, 0.799, respectively (Figure 3C). These metabolites may serve as potential biomarkers to guide early switch to second-line treatment. MetPA identified 5 enriched metabolic pathways perturbed significantly between the two groups in cITP patients (Figure 3D): citrate cycle, pyruvate metabolism, glycolysis/gluconeogenesis, glyoxylate and dicarboxylate metabolism, glycerolipid metabolism (impact value > 0.05 and -log10(p) > 1) (Table 3).

**TABLE 3:** Details of the main metabolic pathways obtained from MetPA in cITP patients.

Pathway name	p	-log(p)	Holm p	Impact value
Citrate cycle (TCA cycle)	0.002301	2.638	0.19331	0.1049
Pyruvate metabolism	0.002788	2.5547	0.23143	0.20684
Glycolysis/Gluconeogenesis	0.003896	2.4094	0.31945	0.10044
Glyoxylate and dicarboxylate metabolism	0.005884	2.2303	0.47072	0.07937
Glycerolipid metabolism	0.060454	1.2186	1	0.09346

### Three Distinct Metabolites and Related Pathways Were Identified Between H. Pylori-Positive and H. Pylori-Negative Patients With cITP

**FIGURE 4:**
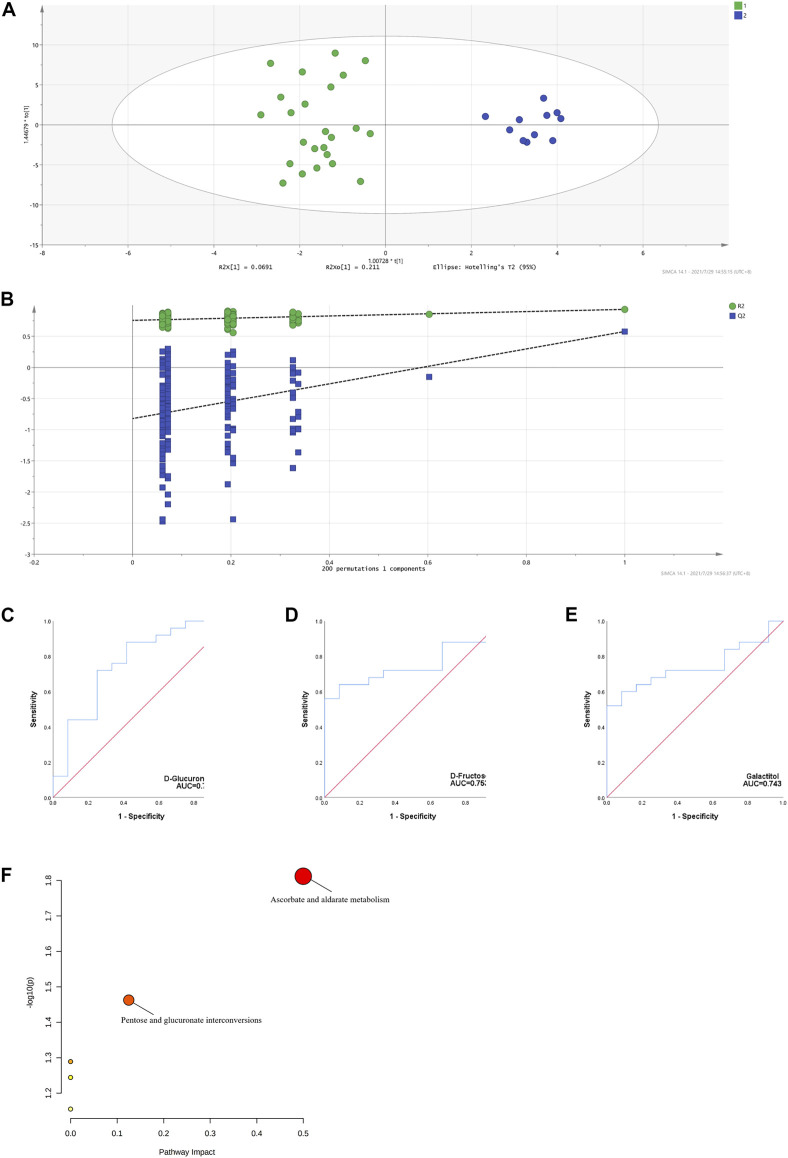
OPLS-DA model based on the data from the cITP patients grouped by **(H)** pylori infection **(A)** score scatter plot **(B)** 200 random permutation tests **(C–E)** ROC of the three distinct metabolites with biomarker potential **(F)** MetPA of the three distinct metabolites in cITP patients grouped by **(H)** pylori infection.

Several studies have demonstrated an increased prevalence of H. pylori infection in patients with cITP (Kohda et al., 2002; Suzuki et al., 2006) and eradication of H. pylori contributed to the increase in platelet counts in cITP patients (Noonavath et al., 2014). Accordingly, we developed the OPLS-DA model based the metabolomic data between H. pylori-positive and H. pylori-negative patients with cITP and validated by permutation tests (Figures 4A,B). Combining the VIP > 1 from OPLS-DA model, *p* < 0.05 and AUC > 0.7 from ROC analysis (Figures 4C–E), three distinct metabolites were eventually obtained: D-fructose, galactitol, D-glucuronic acid. Followed by the MetPA, we revealed the related metabolic pathways were ascorbate and aldarate metabolism, pentose and glucuronate interconversions (Figure 4F).

## Discussion

To the best of our knowledge, no previous research has examined metabolite the comprehensive metabolomic profile of ITP patients or identified the differential plasma metabolites of high diagnostic and prognostic accuracy. In the current study, the metabolomic data acquired by GC-MS discriminated 17 differential metabolites from ITP patients to HCs. Accordingly, the dysregulated metabolic pathways related to ITP included may be associated to phenylalanine, tyrosine and tryptophan biosynthesis, phenylalanine metabolism, glyoxylate and dicarboxylate metabolism.

The mechanism underlying ITP includes enhanced platelet clearance and impaired platelet production, which is mainly ascribe to immune dysregulation mediated by B cells and antibodies, T cell-mediated effects, tolerance checkpoint defects and more (J. Li et al., 2017). However, no study to date has elucidated the pathology of ITP from metabolic disorders. It has been reported that chronic immune stimulation correlates with reduced phenylalanine turnover (Neurauter et al., 2008). Higher serum phenylalanine levels related to immune activation were detectable in patients with Alzheimer’s disease, in the pathogenesis of which immune activation and inflammation represented critical mechanisms (Wissmann et al., 2013). Li et al. suggested that mild immune stress caused metabolic reprogramming including phenylalanine metabolism (Li C.Y. et al., 2020), implicating a potential relationship between immunomodulation and phenylalanine related metabolic pathways. The glyoxylate and dicarboxylate metabolism is closely associated with the citric acid cycle and contributes to the energy metabolism (Song et al., 2021). Previous studies have shown that the shift to glyoxylate and dicarboxylate metabolism is a marker for decreased tolerance to insult (Black et al., 2012). Higher levels of ROS were reported to be involved in the pathogenesis of corticosteroid-resistant ITP(Y. Kong et al., 2018). Cano et al. showed that dysfunctions in glyoxylate and dicarboxylate metabolism pathway indicate mitochondrial dysfunction that would result in decreased ability to detoxify reactive oxygen species (ROS), serving as predisposing factors for the development of therapy-related myelodysplasia syndrome (Cano et al., 2011). Besides, Geng et al. elucidated that glyoxylate and dicarboxylate metabolism contributed to the lipopolysaccharide (LPS)-induced systemic inflammatory response syndrome (SIRS) (Geng et al., 2020) and ITP is commonly considered as an autoimmune disease and regarded as inflammation to some extent (J. Li et al., 2017).

Among the differential metabolites, DHA is the major polyunsaturated fatty acids closely related to platelet activity. DHA has been demonstrated to diminish platelet aggregation and to lengthen bleeding time, while octadecadienoic acid was found to improve platelet aggregation (ten Hoor, 1980) and presented an inverse association with the risk for stroke (Zhang W. et al., 2020). Dietary interventions with DHA may reduce the risk of heart diseases and has been proposed as an antithrombotic therapy (Wijendran and Hayes, 2004), and were found to decrease ROS production and improve platelet redox balance in T2DM patients (McDonald et al., 2013). Increase of DHA was associated with an increased ability of platelets to respond to an inflammatory stimulus (Souza et al., 2020). Enhanced generation of megakaryocytes and platelets was observed in the presence of DHA (Siddiqui et al., 2011), possibly through upregulation of the NOTCH and AKT pathways (Dhenge et al., 2017). In our study, decreased level of DHA was observed in ITP patients compared with HCs, suggesting a profound role of DHA to improve platelets in ITP patients. In our study, glycerol was identified to be the independent risk factor of NSR in ITP patients. Glycerol is a three-carbon substance that forms the backbone of fatty acids in fats. Murgia et al. reported that glycerol was one of the potential biomarkers to distinguish systemic sclerosis from HCs by metabolomics analysis (Murgia et al., 2018). Atorvastatin is a widely used anti-lipid reagent. Emerging studies have indicated that atorvastatin may support megakaryocytopoiesis and exhibited comparable immunomodulatory function, representing a novel therapy approach for corticosteroid-resistant ITP patients. Kong’s study (Y Kong et al., 2018) demonstrated that atorvastatin restored the impaired function of endothelial progenitor cells of corticosteroid-resistant ITP patients and increased the platelet counts of them in a pilot study. More recently, Xu et al. (2021) revealed that atorvastatin ameliorated inhibited the activation of CD4^+^ T cells, leading to the improvement of platelet count. Glycerol monostearate and 1-monopalmitin are both monoacylglycerols, formed biochemically via release of a fatty acid. Monoacylglycerols metabolism is also related to the effects of endocannabinoids in the immune system (Chiurchiu et al., 2015). 3-HB is one of the ketone bodies serving as a biomarker to reflect fatty acid β-oxidation and ketogenic amino acid catabolism (Choi et al., 2020; L.; Guo et al., 2021). 3-HB was also found to be associate with excess glutathione demand and disrupted mitochondrial energy mechanisms, occurring during persistent oxidative stress (Volani et al., 2018). The increased 3-HB production in our study may indicate the greater oxidative stress in ITP patients. As to the involved amino acid metabolism, the glycine level was increased in ITP patients. L-valine is one of the branched-chain amino acids (BCAAs). The BCAA catabolism was reported to regulate platelet activation and was associated with arterial thrombosis risk (Y. Xu et al., 2020). L-proline is a component of collagen metabolites and increased L-proline level may be related to the activation of collagen breakdown (Y. Guo et al., 2020). In addition, glycine was reported to play a role in affecting platelet membrane potential and hence aggregation capacity (Schemmer et al., 2013).

Additionally, we distinguished six significantly altered endogenous metabolites (lactic acid, glyceric acid, L-valine, pyruvic acid, uric acid, α-ketoglutaric acid) and five enriched metabolic pathways (citrate cycle, pyruvate metabolism, glycolysis/gluconeogenesis, glyoxylate and dicarboxylate metabolism, glycerolipid metabolism) in predicting the therapeutic effect of glucocorticoids in chronic ITP patients. Three distinct metabolites (D-fructose, galactitol, D-glucuronic acid) and related pathways (ascorbate and aldarate metabolism, pentose and glucuronate interconversions) were identified between H. pylori-positive and H. pylori-negative patients with cITP. The dysregulated metabolic pathways were mainly associated with glucose metabolism, which is essential for platelet function and clearance (Fidler et al., 2017). However, the exact mechanisms between these identified metabolic profiles with ITP are far from clear. As limitations of sample qualities, more patients need to be included in the future study.

## Conclusion

In conclusion, this study, using GC-MS based metabolomic methods, has provided for the first time a comprehensive metabolomic profile in plasma of ITP patients. Our explanation of the metabolic disturbance described here and the underlying mechanisms are far from clear. The exact relationships between these metabolic alterations and ITP development need to be validated subsequently by researches with a larger sample size.

## Data Availability

The original contributions presented in the study are included in the article/Supplementary Material, further inquiries can be directed to the corresponding author.
