# Supplementary material for: Plasma Metabolomics Identifies the Dysregulated Metabolic Profile of Primary Immune Thrombocytopenia (ITP) Based on GC-MS
Source: Front Pharmacol. 2022 May 24;13:845275. doi: 10.3389/fphar.2022.845275 (PMC9170960; doi:10.3389/fphar.2022.845275)
Supplement: Supplementary file 1 [file DataSheet1.PDF]

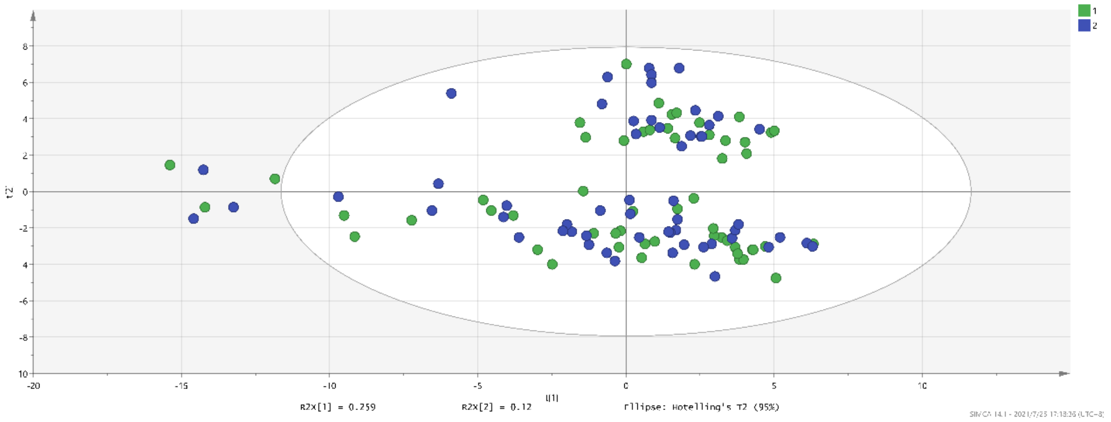

Supplementary Figure 1. Score scatter plot of the plasma metabolites in ITP patients before and after treatment

Supplementary Table 1. Propensity Score Matching of the subjects

patient_id	group	sex	age	u	_pscore	_weight	_id	_n1	_n2	_nn	_pdif
1	0	1	50	0.428	0.769	3.000	19	78	79	2	0.0000
2	0	0	39	0.450	0.778	4.000	20	82	83	2	0.0000
3	0	0	26	0.314	0.746	3.500	11	57	58	4	0.0000
4	0	1	40	0.386	0.744	3.000	10	54	55	4	0.0000
5	0	1	14	0.687	0.672	2.000	1	30	31	2	0.0000
6	0	1	26	0.487	0.706	1.000	4	35	36	2	0.0055
7	0	0	30	0.672	0.756	2.000	15	68	69	4	0.0025
8	0	0	44	0.673	0.789	5.500	23	90	91	4	0.0000
9	0	1	42	0.188	0.749	3.000	12	60	61	5	0.0001
10	0	0	52	0.164	0.807	4.000	27	100	101	5	0.0000
11	0	1	60	0.905	0.792	1.500	24	94	95	2	0.0000
12	0	0	44	0.575	0.789	2.500	22	90	91	4	0.0000
13	0	0	56	0.314	0.815	12.000	28	108	107	2	0.0020
14	0	0	25	0.770	0.744	2.833	9	53	54	2	0.0000
15	0	1	21	0.665	0.692	2.500	2	34	33	2	0.0029
16	0	0	78	0.047	0.855		129				
17	0	1	48	0.764	0.764	4.167	18	74	75	3	0.0000
18	0	0	51	0.699	0.805	1.000	25	100	101	5	0.0021
19	0	1	48	0.218	0.764	1.333	17	74	75	3	0.0000
20	0	0	14	0.402	0.715	3.500	5	36	35	2	0.0000
21	0	0	21	0.926	0.733	6.500	6	46	47	3	0.0000
22	0	0	29	0.408	0.754	1.667	13	66	67	3	0.0000
23	0	0	42	0.807	0.785	3.000	21	87	88	2	0.0000
24	0	1	26	0.025	0.706	0.500	3	35	36	2	0.0055
25	0	0	25	0.326	0.744	1.667	8	53	54	2	0.0000
26	0	0	31	0.606	0.759	2.000	16	68	69	4	0.0000
27	0	0	61	0.486	0.825	10.500	29	114	115	4	0.0000
28	0	0	29	0.906	0.754	1.333	14	66	67	3	0.0000
29	0	0	51	0.924	0.805	4.500	26	100	101	5	0.0021
30	0	0	23	0.839	0.739	5.000	7	50	51	2	0.0000
31	1	1	16	0.309	0.678		32	1	2	2	0.0059
32	1	0	42	0.445	0.785	0.500	87	21	22	2	0.0000
33	1	0	27	0.830	0.749	0.200	62	12	11	2	0.0001
34	1	0	52	0.092	0.807	0.600	100	27	26	2	0.0000
35	1	0	44	0.778	0.789	1.000	91	22	23	3	0.0000
36	1	1	64	0.883	0.800		98	25	26	3	0.0042
37	1	0	36	0.182	0.771	0.500	79	19	20	2	0.0023
38	1	0	21	0.498	0.733	0.667	47	6	7	2	0.0000
39	1	0	63	0.407	0.829		120	29	28	2	0.0038
40	1	1	59	0.669	0.789		93	22	23	3	0.0000
41	1	1	38	0.906	0.739	0.500	51	7	8	2	0.0001
42	1	1	40	0.830	0.744	0.250	56	10	9	2	0.0000

43	1	1	48	0.731	0.764	0.667	74	17	18	3	0.0000
44	1	0	50	0.655	0.802		99	25	26	3	0.0021
45	1	1	49	0.867	0.766		77	19	18	2	0.0024
46	1	1	16	0.013	0.678	0.500	31	1	2	2	0.0059
47	1	0	62	0.966	0.827		118	29	28	2	0.0019
48	1	0	27	0.432	0.749	0.400	61	12	11	2	0.0001
49	1	0	40	0.835	0.780		85	20	21	2	0.0023
50	1	0	24	0.932	0.741		52	8	9	3	0.0026
51	1	0	65	0.830	0.832		126	29	28	2	0.0076
52	1	0	25	0.802	0.744	1.000	53	8	9	3	0.0000
53	1	0	19	0.177	0.728		42	6	7	2	0.0053
54	1	1	28	0.136	0.712	1.500	35	5	4	2	0.0027
55	1	0	62	0.588	0.827		117	29	28	2	0.0019
56	1	1	52	0.068	0.773		81	20	19	2	0.0046
57	1	1	19	0.529	0.687	0.500	33	2	1	2	0.0057
58	1	0	60	0.670	0.823		112	29	28	2	0.0019
59	1	0	44	0.996	0.789	0.500	92	22	23	3	0.0000
60	1	1	54	0.909	0.778		84	20	21	2	0.0001
61	1	0	65	0.578	0.832		125	29	28	2	0.0076
62	1	0	17	0.950	0.723		39	5	6	2	0.0081
63	1	0	55	0.811	0.813	0.500	107	28	27	2	0.0020
64	1	0	31	0.023	0.759	0.500	68	16	15	2	0.0000
65	1	0	53	0.249	0.809		104	27	26	2	0.0021
66	1	0	65	0.523	0.832		124	29	28	2	0.0076
67	1	0	39	0.042	0.778	0.500	82	20	21	2	0.0000
68	1	1	50	0.002	0.769	0.500	78	19	18	2	0.0000
69	1	1	40	0.628	0.744	0.500	55	10	9	2	0.0000
70	1	0	65	0.867	0.832		127	29	28	2	0.0076
71	1	0	52	0.752	0.807	0.600	102	27	26	2	0.0000
72	1	0	31	0.108	0.759	1.000	69	16	15	2	0.0000
73	1	0	61	0.527	0.825	0.500	115	29	28	2	0.0000
74	1	0	55	0.297	0.813		106	28	27	2	0.0020
75	1	0	61	0.400	0.825	0.250	114	29	28	2	0.0000
76	1	0	63	0.693	0.829		122	29	28	2	0.0038
77	1	0	41	0.411	0.783		86	21	20	2	0.0023
78	1	0	60	0.939	0.823		113	29	28	2	0.0019
79	1	0	66	0.282	0.834		128	29	28	2	0.0095
80	1	0	21	0.378	0.733	0.333	46	6	7	2	0.0000
81	1	1	61	0.854	0.794		96	24	23	2	0.0022
82	1	1	46	0.706	0.759		71	16	15	2	0.0001
83	1	1	58	0.256	0.787		89	22	23	3	0.0022
84	1	0	26	0.141	0.746	0.500	58	11	10	2	0.0000
85	1	0	22	0.547	0.736		48	7	6	2	0.0026
86	1	1	32	0.381	0.723		40	5	6	2	0.0082

87	1	0	57	0.214	0.817	0.500	108	28	29	2	0.0020
88	1	1	12	0.049	0.666		130				
89	1	1	35	0.560	0.731		45	6	7	2	0.0026
90	1	0	31	0.345	0.759	0.500	70	16	15	2	0.0000
91	1	0	36	0.606	0.771		80	19	20	2	0.0023
92	1	0	29	0.035	0.754	0.667	66	13	14	3	0.0000
93	1	0	28	0.135	0.751		64	12	13	2	0.0025
94	1	0	52	0.486	0.807	1.200	101	27	26	2	0.0000
95	1	0	46	0.243	0.794	0.500	95	24	23	2	0.0022
96	1	1	22	0.437	0.695	0.500	34	2	3	2	0.0029
97	1	0	63	0.522	0.829		121	29	28	2	0.0038
98	1	1	64	0.282	0.800		97	25	26	3	0.0042
99	1	0	44	0.283	0.789	0.500	90	22	23	3	0.0000
100	1	0	16	0.583	0.720		37	5	6	2	0.0054
101	1	0	63	0.291	0.829		119	29	28	2	0.0038
102	1	0	22	0.911	0.736		49	7	6	2	0.0026
103	1	0	58	0.044	0.819		110	28	29	2	0.0040
104	1	1	49	0.099	0.766		76	19	18	2	0.0024
105	1	1	72	0.829	0.817		109	28	29	2	0.0021
106	1	0	64	0.126	0.831		123	29	28	2	0.0057
107	1	0	29	0.280	0.754	1.333	67	13	14	3	0.0000
108	1	0	14	0.329	0.715	1.500	36	5	4	2	0.0000
109	1	0	17	0.251	0.723		38	5	6	2	0.0081
110	1	1	33	0.427	0.726		41	6	5	2	0.0079
111	1	1	54	0.234	0.778	0.500	83	20	21	2	0.0001
112	1	0	28	0.429	0.751		65	12	13	2	0.0025
113	1	0	45	0.611	0.792	0.500	94	24	23	2	0.0000
114	1	1	40	0.447	0.744	1.250	54	10	9	2	0.0000
115	1	1	48	0.875	0.764	1.333	75	17	18	3	0.0000
116	1	1	14	0.859	0.672	0.500	30	1	2	2	0.0000
117	1	0	26	0.013	0.746	0.250	57	11	10	2	0.0000
118	1	0	33	0.177	0.764		72	17	18	3	0.0001
119	1	0	27	0.954	0.749	0.200	63	12	11	2	0.0001
120	1	0	61	0.829	0.825	0.250	116	29	28	2	0.0000
121	1	0	54	0.356	0.811		105	28	27	2	0.0041
122	1	0	27	0.276	0.749	0.200	60	12	11	2	0.0001
123	1	0	43	0.908	0.787	0.500	88	22	23	3	0.0022
124	1	0	26	0.397	0.746	0.250	59	11	10	2	0.0000
125	1	0	19	0.389	0.728		43	6	7	2	0.0053
126	1	0	33	0.867	0.764		73	17	18	3	0.0001
127	1	0	20	0.802	0.731		44	6	7	2	0.0026
128	1	0	23	0.972	0.739	0.500	50	7	8	2	0.0000
129	1	0	52	0.899	0.807	0.600	103	27	26	2	0.0000
130	1	0	60	0.047	0.823		111	29	28	2	0.0019

Supplementary Table 2. Data of the differential metabolites obtained from univariate and multivariate OPLS-DA

Peaks(mz/rt)	VIP value	t.stat	p.value	FDR
Myo-Inositol	1.56401	-3.9506	0.00028514	0.0098004
Glycerol monostearate	1.5092	-3.7017	0.00060501	0.0098004
1-Monopalmitin	1.46536	-3.5356	0.00098831	0.0098004
Docosahexaenoic acid	1.45497	-3.5024	0.0010889	0.0098004
L-Threonic acid	1.22501	-3.4202	0.0013821	0.0099511
3-Hydroxybutyric acid	1.41126	3.0962	0.003444	0.014227
Cis-aconitate	1.36913	-3.0614	0.0037895	0.014227
L-Valine	1.13619	3.0563	0.0038421	0.014227
Octadecadienoic acid	1.38532	3.0522	0.003886	0.014227
2-Methylhexanoic Acid	1.40632	3.046	0.0039521	0.014227
Glycine	1.29592	3.0084	0.004378	0.014328
3-hydroxypropionic acid	1.33237	-2.9376	0.0052979	0.015894
Glycerol	1.27819	-2.7651	0.0083483	0.021975
Phenylalanine	1.28737	-2.7561	0.0085458	0.021975
Galactitol	1.183	2.5923	0.012975	0.029379
L-Leucine	1.22052	-2.5898	0.013057	0.029379
Creatinine	1.21628	-2.4269	0.019493	0.035873
D-Fructose	1.14342	2.417	0.019962	0.035873
L-Proline	1.1904	-2.3668	0.022517	0.035873
Isobutyric acid	1.20741	2.3584	0.022969	0.035873
L-Isoleucine	1.14901	-2.3506	0.0234	0.035873
α-ketoglutaric acid	1.03234	-2.3376	0.02413	0.035873
Myo-Inositol	1.09483	-2.3359	0.024227	0.035873
Glyceric acid	1.19464	-2.3264	0.024777	0.035873
Xylitol	1.12547	-2.3127	0.025593	0.035873
L-Tyrosine	1.22501	-2.3074	0.025908	0.035873
Succinic Acid	1.12604	-2.2608	0.028888	0.037765
L-Threonine	1.09573	-2.2407	0.030267	0.037765
Oxalic acid	1.11955	-2.2384	0.030422	0.037765
Stearic acid	1.15173	2.2123	0.032307	0.038769
Isovaleric acid	1.04245	-2.1484	0.037354	0.043378

Supplementary Table 3. AUC of the 17 differential metabolites with biomarker potential in ITP in the test set

Num	Metabolites	AUC	p value
1	Glycerol monostearate	0.869	<0.001
2	Cis-aconitate	0.868	<0.001
3	Docosahexaenoic acid	0.847	<0.001
4	Glycerol	0.835	<0.001
5	1-Monopalmitin	0.828	<0.001
6	Myo-Inositol	0.802	<0.001
7	L-Valine	0.801	<0.001
8	Octadecadienoic acid	0.788	0.001
9	3-Hydroxybutyric acid	0.786	0.001
10	Phenylalanine	0.778	0.001
11	3-hydroxypropionic acid	0.773	0.001
12	Creatinine	0.773	0.001
13	D-Fructose	0.767	0.001
14	Isovaleric acid	0.760	0.002
15	Galactitol	0.757	0.002
16	L-Proline	0.742	0.003
17	Glycine	0.718	0.008
